# Supplementary material for: MetaQTL: a package of new computational methods for the meta-analysis of QTL mapping experiments
Source: BMC Bioinformatics. 2007 Feb 8;8:49. doi: 10.1186/1471-2105-8-49 (PMC1808479; doi:10.1186/1471-2105-8-49)
Supplement: Additional File 7 — MetaQTL Package : jar file and tutorial. This Zip archive contains both the MetaQTL JAR file and the files of the tutorial. [file 1471-2105-8-49-S7.zip › org.inra.metaqtl/doc/org/metaqtl/graph/QtlLayer.html]

QtlLayer


|  |  |  |
| --- | --- | --- |
| **Overview** \| **Package** \| **Class** \| **Use** \| **Tree** \| **Deprecated** \| **Index** \| **Help** | |  |
| **PREV CLASS**   **NEXT CLASS** | **FRAMES**    **NO FRAMES**     **All Classes** |  |
| SUMMARY: NESTED \| FIELD \| CONSTR \| METHOD | DETAIL: FIELD \| CONSTR \| METHOD |  |


---


## org.metaqtl.graph Class QtlLayer

```
java.lang.Object
  org.metaqtl.graph.AnchoredElement
      org.metaqtl.graph.Layer
          org.metaqtl.graph.QtlLayer
```

---

``` public class QtlLayer extends Layer ```

---

| **Field Summary** | |
| --- | --- |
| `static double` | `CI_WIDTH`             The default width of the CI of the Qtl |
| `QtlShape[]` | `qtlShapes`             The Qtl shapes. |
| `static double` | `TEXT_WIDTH`             The default width of the Text attribute of the Qtl |
| `static double` | `TICK_CEX_HEIGHT`             The default cex parameter for the tick height of the Qtl |
| `static double` | `TICK_CEX_WIDTH`             The default cex parameter for the tick width of the Qtl |

**Fields inherited from class org.metaqtl.graph.Layer**

`height, width`

**Fields inherited from class org.metaqtl.graph.AnchoredElement**

`x, y`


**Constructor Summary**

`QtlLayer(double x,
double y)`


| **Method Summary** | |
| --- | --- |
| `void` | `attach(java.lang.Object object)`             Attach the given `object` to the layer. |
| `void` | `build(java.awt.Graphics2D graph)`             Build the layer. |
| `void` | `draw(java.awt.Graphics2D graph)`             Draws the layer on the given `graph` context. |
| `double` | `getYMax()` |
| `double` | `getYMin()` |

**Methods inherited from class org.metaqtl.graph.Layer**

`getHeight, getWidth`

**Methods inherited from class org.metaqtl.graph.AnchoredElement**

`getX, getY, setX, setY`

**Methods inherited from class java.lang.Object**

`clone, equals, finalize, getClass, hashCode, notify, notifyAll, toString, wait, wait, wait`

**Field Detail**

### CI\_WIDTH

```
public static double CI_WIDTH
```

:   The default width of the CI of the Qtl

---


### TICK\_CEX\_HEIGHT

```
public static double TICK_CEX_HEIGHT
```

:   The default cex parameter for the tick height
    of the Qtl

---


### TICK\_CEX\_WIDTH

```
public static double TICK_CEX_WIDTH
```

:   The default cex parameter for the tick width
    of the Qtl

---


### TEXT\_WIDTH

```
public static double TEXT_WIDTH
```

:   The default width of the Text attribute of the
    Qtl

---


### qtlShapes

```
public QtlShape[] qtlShapes
```

:   The Qtl shapes.


**Constructor Detail**

### QtlLayer

```
public QtlLayer(double x,
                double y)
```

**Parameters:**: `x` -: `y` -: `size` -: `type` -


**Method Detail**

### draw

```
public void draw(java.awt.Graphics2D graph)
```

:   **Description copied from class: `Layer`**
:   Draws the layer on the given `graph` context.

    :   **Specified by:**: `draw` in class `Layer`

---


### build

```
public void build(java.awt.Graphics2D graph)
```

:   **Description copied from class: `Layer`**
:   Build the layer.

    :   **Specified by:**: `build` in class `Layer`

---


### attach

```
public void attach(java.lang.Object object)
```

:   **Description copied from class: `Layer`**
:   Attach the given `object` to the layer.

    :   **Specified by:**: `attach` in class `Layer`
    :   **Parameters:**: `object` - the object to attach to the layer.

---


### getYMin

```
public double getYMin()
```

:   **Specified by:**: `getYMin` in class `Layer`

---


### getYMax

```
public double getYMax()
```

:   **Specified by:**: `getYMax` in class `Layer`


---


|  |  |  |
| --- | --- | --- |
| **Overview** \| **Package** \| **Class** \| **Use** \| **Tree** \| **Deprecated** \| **Index** \| **Help** | |  |
| **PREV CLASS**   **NEXT CLASS** | **FRAMES**    **NO FRAMES**     **All Classes** |  |
| SUMMARY: NESTED \| FIELD \| CONSTR \| METHOD | DETAIL: FIELD \| CONSTR \| METHOD |  |


---
